# Spicatoside A derived from *Liriope platyphylla* root ethanol extract inhibits hepatitis E virus genotype 3 replication *in vitro*

**DOI:** 10.1038/s41598-019-39488-5

**Published:** 2019-03-13

**Authors:** Gayoung Park, Amna Parveen, Jung-Eun Kim, Kyo Hee Cho, Sun Yeou Kim, Bang Ju Park, Yoon-Jae Song

**Affiliations:** 1 Department of Life Science, Gachon University, Seongnam-Si, 13120 Korea; 2 College of Pharmacy, Gachon University, Incheon, 21936 Korea; 3 Gachon Institute of Pharmaceutical Science, Gachon University, Incheon, 21936 Korea; 4 Department of Pharmacognosy, Faculty of Pharmaceutical Science, Government College University, Faisalabad, Pakistan; 5 Department of Electronic Engineering, Gachon University, Seongnam-Si, 13120 Korea

## Abstract

Hepatitis E virus (HEV) is the causative agent of hepatitis E in humans worldwide. Although hepatitis E is self-limiting without chronic infection development, HEV infection often leads to severe liver diseases causing high mortality in pregnant women in addition to chronic hepatitis and cirrhosis in immunosuppressed patients. In this study, we investigated the effect of a *Liriope platyphylla* ethanol extract (LPE) on HEV replication. Interestingly, LPE suppressed replication of the genotype 3 HEV replicon. Sequential solvent fractionation revealed that the ethyl acetate (EA) fraction of LPE exerts the most potent inhibitory effects. With the aid of activity-guided fractionation and multi-step column chromatography, spicatoside A was subsequently isolated in the EA fraction of LPE and specifically shown to exert inhibitory effects on replication of the genotype 3 HEV replicon. In addition, spicatoside A interfered with replication of the HEV genotype 3 strain 47832c and expression of HEV ORF2 capsid proteins. Our findings clearly support the potential utility of spicatoside A as an effective anti-HEV agent.

## Introduction

Hepatitis E virus (HEV), a member of the *Hepeviridae* family transmitted via the fecal-oral route, is the causative agent of hepatitis E^1^. The virus has a single-stranded, positive-sense RNA genome 7.2 kb in size with a capped 5′ end and polyadenylated 3′ end^2,3^.

The HEV genome contains three open reading frames (ORFs) designated ORF1, 2 and 3^2^. ORF1 encodes a non-structural replicase polyprotein with several functional domains including methyltransferase (Met), Y domain, papain-like cysteine protease (PCP), hypervariable region (HVR), X-domain, helicase domain (Hel) and RNA dependent RNA polymerase (RdRp)^4^. ORF2 encodes the capsid protein that binds cellular proteins, such as heparin sulfate proteoglycan (HSPG), heat-shock protein 90 (HSP90) and glucose-regulated protein 78 (Grp78) while ORF3 encodes a multifunctional phosphoprotein important for release of the HEV virion^5–8^. In addition, HEV ORF3 is reported to inhibit the host innate immune response by degrading tumor necrosis factor receptor 1-associated death domain (TRADD) protein, reducing ubiquitination of the receptor interacting protein 1 (RIP1) and suppressing NF-κB activation^9^.

After entry into the host cell, viral genomic RNA serves as an mRNA for ORF1 translation and a template for replication. The viral genomic RNA translates ORF1 protein, and the largest ORF1 domain, RdRp, synthesizes negative-sense RNA which is used as a template for subsequent synthesis of positive-sense genomic and subgenomic RNAs^4^. ORF2 and ORF3 are translated from subgenomic RNA, and viral genomic RNA is incorporated into the virion.

HEV isolates infecting humans belong to the *Orthohepevirus* A species^10–13^. Within *Orthohepevirus A*, four genotypes of HEV are implicated in human infection to date^11,13^. Genotypes 1 and 2 mainly exist in developing countries. Genotype 1 has been detected in Asia and Africa, and genotype 2 in Mexico and Africa with infections by both genotypes being limited to humans. In contrast, genotypes 3 and 4 are primarily zoonotic. Several animal species, including pigs, wild boar, deer and mongoose, serve as reservoirs of HEV. Genotype 3 has been detected not only in developing but also developed countries, such as Japan and the United States, and the European continent while genotype 4 is more prevalent in Asia^12,13^. In addition to genotypes 1–4, a human case of genotype 7 infection is reported^14^.

Approximately 20 million people worldwide are infected with HEV^15^. Despite its self-limiting nature, HEV infection can trigger a serious liver disease that is fatal in up to 3.3% cases^15^. More importantly, HEV infection can cause fulminant hepatic failure in pregnant women with an estimated mortality rate of ~30%^16,17^. In addition, HEV genotype 3 is reported to cause chronic viral hepatitis and liver cirrhosis in organ transplant recipients on immunosuppressive therapy^18^.

Currently, no specific treatments are available for HEV infection. Ribavirin and interferon α (IFN-α) are generally used to treat immunosuppressed or immunocompromised patients with chronic hepatitis E^19^. Both ribavirin and IFN-α efficiently suppress HEV but induce long-term side effects^20^. Attempts to develop novel antivirals against HEV infection have been reported^21,22^. Anang *et al*. showed that a cyclic peptide inhibitor blocks HEV release from the infected cells by preventing interactions between HEV ORF3 and the host tumor susceptibility gene 101^23^. Zinc salts have been demonstrated to inhibit HEV RdRP activity^24^. Another recent study indicated that natural compound silvestrol inhibits HEV replication^25^.

The plant species *Liriope platyphylla* is widely employed as a herbal medicine to treat various chronic diseases, such as diabetes and inflammation, in Korea and China and additionally is reported to possess antiviral activity against hepatitis B virus (HBV)^26–29^. In the present study, we investigated the potential antiviral effects of a 70% ethanol extract of *L. platyphylla* (LPE) and derived bioactive compound(s) on HEV genotype 3 replication.

## Results

### LPE inhibits replication of the HEV genotype 3 replicon

**Figure 1:**
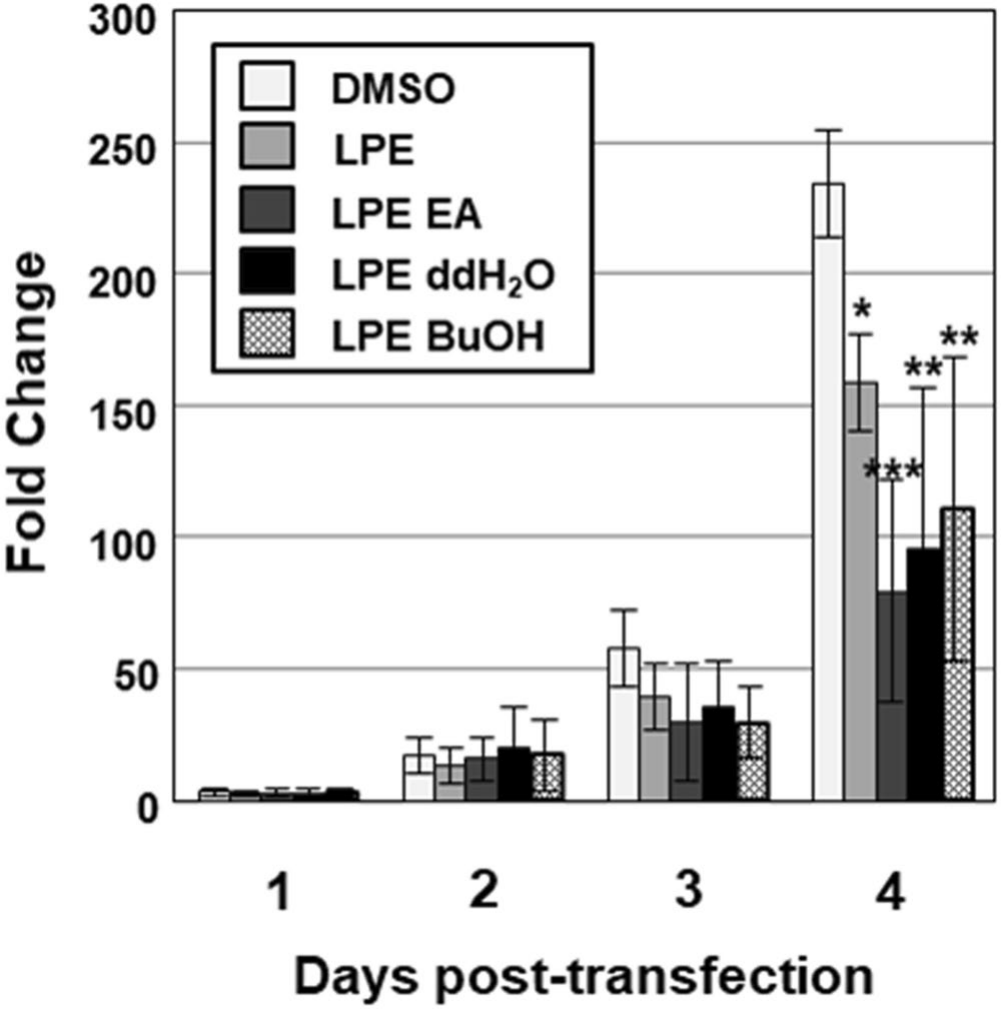
LPE inhibits replication of the HEV luciferase replicon. Huh7.5 cells were mock-transfected or transfected with capped RNA transcripts from the pSHEV3-luc replicon and luc-pcDNA3. After incubation at 37 °C for 5 h, cells were treated with DMSO (vehicle control), LPE or solvent fractions of LPE (EA, *n*-BuOH or ddH_2_O) at a concentration of 10 μg/ml. Cells were re-treated with DMSO, LPE or LPE fractions at 3 d after transfection. At 1, 2, 3 and 4 days after treatment, luciferase activity was determined using a dual luciferase assay system. Luciferase activity of the pSHEV3-luc replicon was expressed as relative light units (RLU) by normalizing *Renilla* luciferase activity to the constitutive firefly luciferase activity of luc-pcDNA3 transcripts. Relative luciferase activity was calculated by reference to luciferase activity of the pSHEV3-luc replicon in the presence of DMSO 1 d after transfection and defined as 1. *P*-values were determined by unpaired two-tailed Student’s *t*-tests (significant at *P < 0.05; **P < 0.01, ***P < 0.001). The data shown here represent four independent experiments.

Huh7.5 cells were transfected with transcripts from the pSHEV3-luc replicon and subsequently treated with 10 μg/ml LPE. Four days after transfection, luciferase activity of the pSHEV3-luc replicon was increased 234.7-fold in control (DMSO-treated) cells (Fig. 1). The increase in luciferase activity in LPE-treated cells was significantly lower, up to a value of 67% that in control cells (Fig. 1). To identify the specific solvent fraction of LPE responsible for inhibiting replication of the pSHEV3-luc replicon, LPE was subjected to sequential extraction with ethyl acetate (EA), butanol (*n*-BuOH) and ddH_2_O. Huh7.5 cells transfected with transcripts from the pSHEV3-luc replicon were treated with the EA, *n*-BuOH or ddH_2_O fraction of LPE (10 μg/ml), and luciferase activity measured 1, 2, 3 and 4 d after transfection. Interestingly, solvent fractions of LPE inhibited replication more effectively than LPE (Fig. 1). Specifically, treatment of cells with the EA, *n*-BuOH and ddH_2_O fractions reduced luciferase activity of the pSHEV3-luc replicon to 33.8%, 47.2% and 40.8%, respectively, relative to that in control DMSO-treated cells (Fig. 1).

### The EA fraction of LPE inhibits replication of HEV genotype 3 strain 47832c *in vitro*

**Figure 2:**
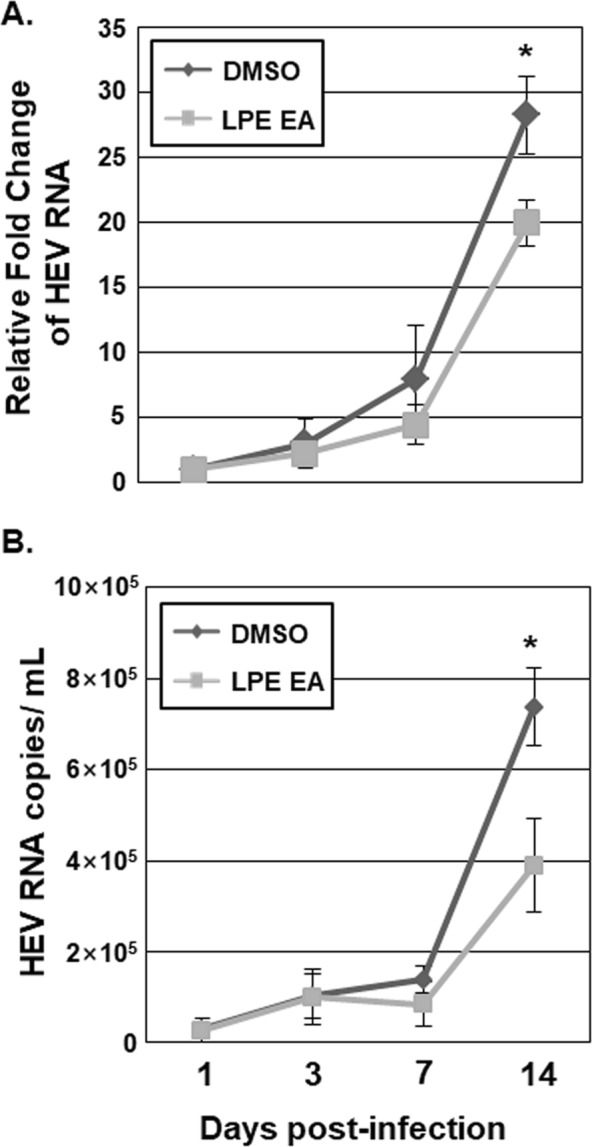
The EA fraction of LPE inhibits replication of the HEV genotype 3 strain 47832c. A549 cells were inoculated with cell culture supernatants containing HEV genotype 3 strain 47832c and incubated at room temperature for 1 h. Cells were treated with either DMSO or the EA fraction of LPE and re-treated again with either DMSO or the EA fraction every 3 d after the initial treatment. At 1, 3, 7 and 14 d after inoculation, (**A**) the relative amounts of HEV RNA in cells and (**B**) the amounts of HEV RNA copies in culture media were determined via qRT-PCR using primers specific for HEV ORF1. *P*-values were determined by unpaired two-tailed Student’s *t*-tests (significant at *P < 0.05). Relative amounts of cell-associated HEV RNA in the presence of DMSO 1 d after inoculation was defined as 1. The data shown here represent three independent experiments.

To ascertain the inhibitory effects of the EA fraction of LPE against HEV replication, we inoculated A549 cells with HEV genotype 3 strain 47832c, followed by treatment with the EA fraction. At 1, 3, 7 and 14 d after inoculation, HEV copy numbers in cells (cell-associated HEV RNA) and culture media (cell-free HEV RNA) were estimated using qRT-PCR (Fig. 2). At 14 d after inoculation, the amount of HEV RNA in cells was increased 28.3-fold in DMSO-treated control cells (Fig. 2A). In cells treated with the EA fraction, the increase in HEV RNA was 70% lower than that in the DMSO treatment group (Fig. 2A). In parallel, the increase in HEV RNA in the culture media of cells treated with the EA fraction was 52.8% lower than that in the DMSO treatment group (Fig. 2B).

### The EA fraction of LPE exerts no significant cytotoxic effects against Huh7.5 or A549 cells

**Figure 3:**
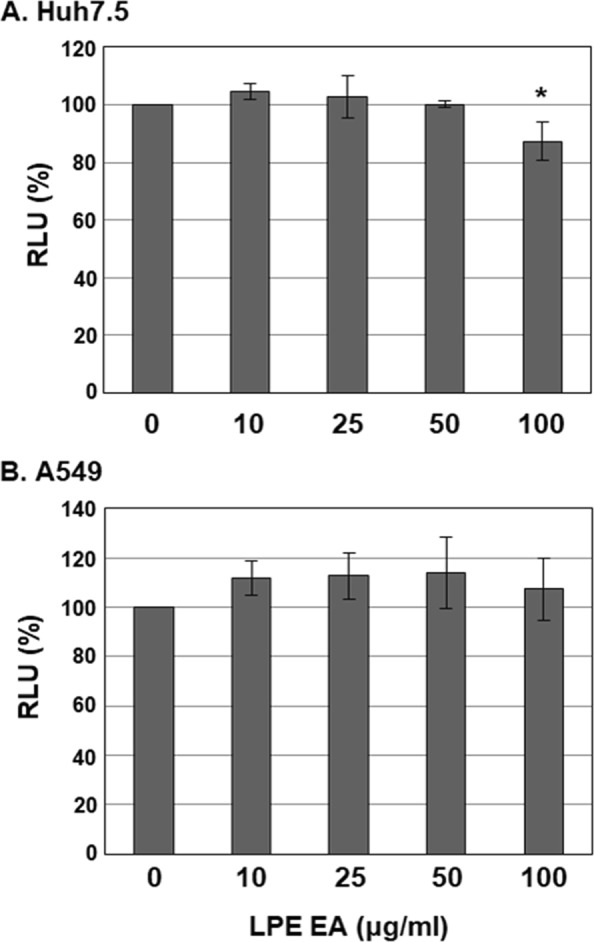
Effects of the EA fraction of LPE on cell viability. (**A**) Huh7.5 or (**B**) A549 cells were treated with varying concentrations of the EA fraction of LPE (0, 10, 25, 50 and 100 µg/ml). Cells were re-treated with the EA fraction every 3 d after the initial treatment. Huh7.5 and A549 cell viability was determined at 4 and 14 d, respectively, after treatment using the CellTiter-Glo Luminescent Cell Viability Assay. Relative luciferase activity was calculated by reference to RLU of cells treated with 0 µg/ml LPE and defined as 100%. *P*-values were determined by paired two-tailed Student’s *t*-tests (significant at *P < 0.05). The data shown here represent four independent experiments.

To examine the possibility that the inhibitory effects of the EA fraction of LPE on HEV replication are mediated via cytotoxic effects, Huh7.5 and A549 cells were treated with different concentrations of 0, 10, 25, 50 and 100 µg/ml EA and cell viability assessed. During the incubation period, cells were re-treated with the EA fraction of LPE every 3 d after the initial treatment. Notably, after a 4-d treatment period, the EA fraction of LPE exerted no significant cytotoxic effects against Huh7.5 cells, except at the highest concentration (100 µg/ml) where Huh7.5 cell viability was only slightly reduced (87%) (Fig. 3A). In A549 cells, the EA fraction had no cytotoxic effects up to the maximum concentration tested (100 µg/ml) after 14 d treatment (Fig. 3B). As previously reported, HEV genotype 3 strain 47832c infection had no cytotoxic effects (data not shown)^30^. Furthermore, HEV genotype 3 strain 47832c infection exerted no additional or synergistic cytotoxic effects against A549 cells with the EA fraction of LPE (data not shown). These findings indicate that the antiviral effect of the EA fraction of LPE against HEV is not attributable to cytotoxicity.

### Identification of Spicatoside A

**Figure 4:**
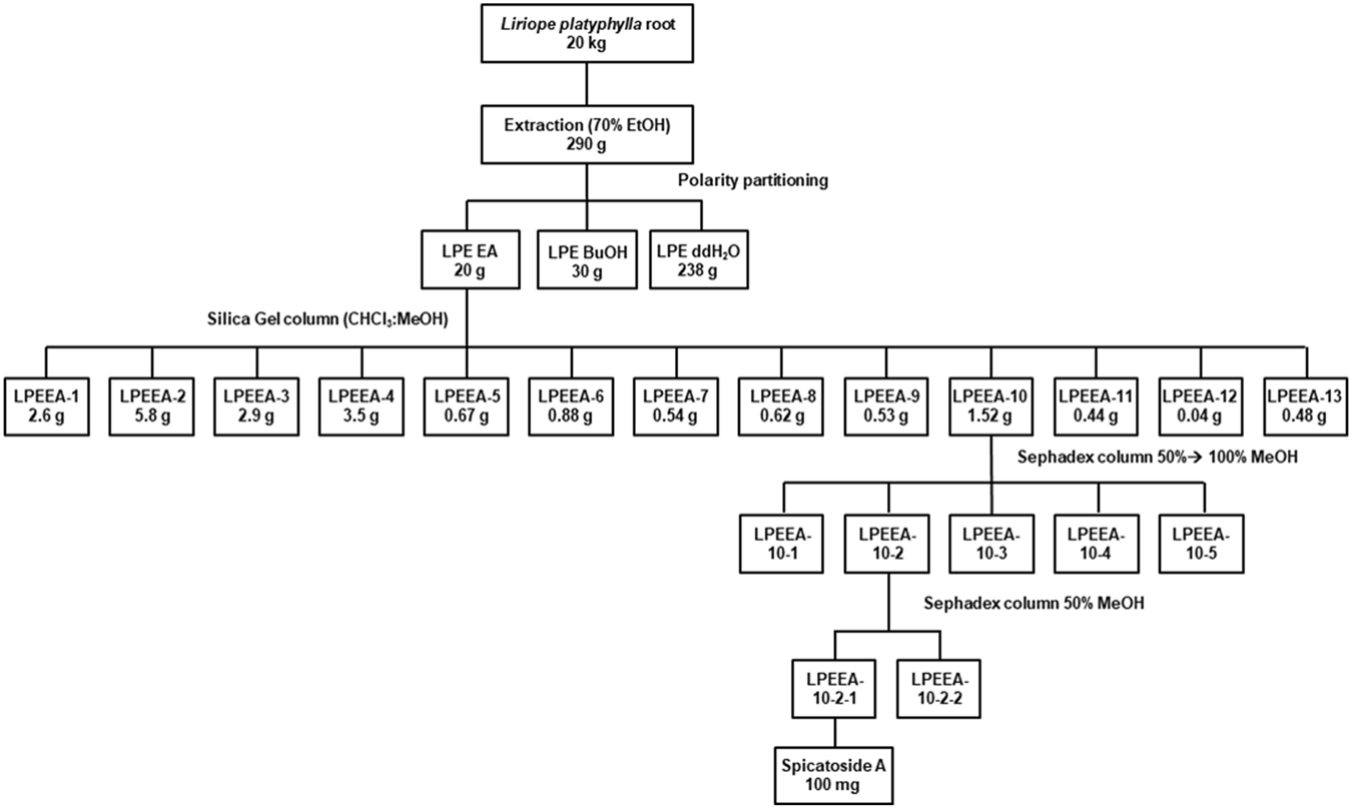
Isolation scheme of spicatoside A derived from *Liriope platyphylla* root.

**Figure 5:**
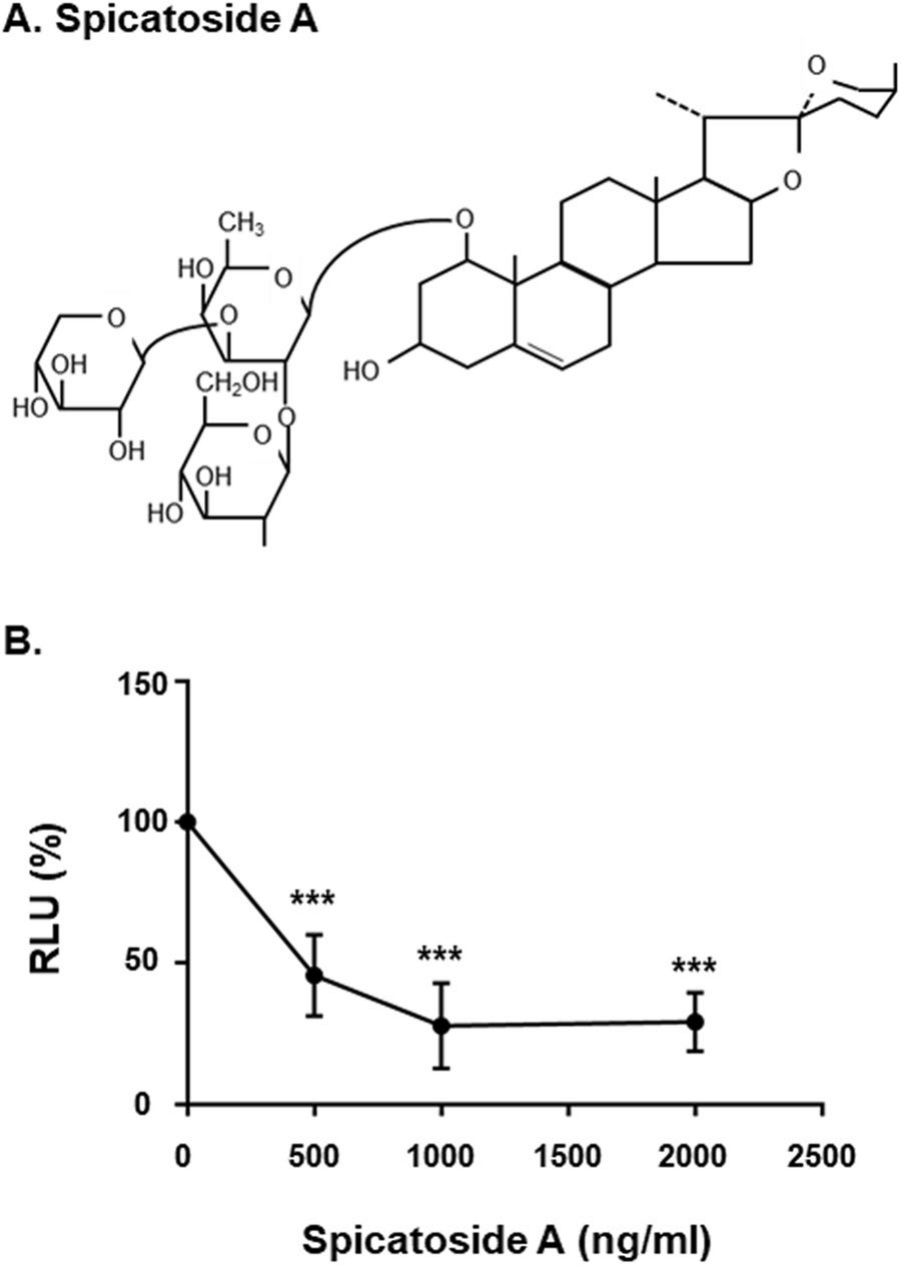
Determination of the anti-HEV effect of spicatoside A. (**A**) Spicatoside A structure. (**B**) Concentration-dependent inhibitory effects of spicatoside A on luciferase activity of the pSHEV3-luc replicon. Huh7.5 cells were mock-transfected or transfected with capped RNA transcripts from the pSHEV3-luc replicon and luc-pcDNA3. After incubation at 37 °C for 5 h, cells were treated with either DMSO or spicatoside A at concentrations of 0, 0.5, 1, and 2 µg/ml. Cells were re-treated with spicatoside A every 3 d after the initial treatment. At 4 d after treatment, luciferase activity was determined using a dual luciferase assay system. The luciferase activity of the pSHEV3-luc replicon was expressed in RLU by normalizing *Renilla* luciferase activity to constitutive firefly luciferase activity of luc-pcDNA3 transcripts. Relative luciferase activity was calculated by reference to luciferase activity of the pSHEV3-luc replicon in the presence of DMSO 4 d after transfection and defined as 100. *P*-values were determined by unpaired two-tailed Student’s *t*-tests (significant at ***P < 0.001). The data shown here represent six independent experiments.

To determine the chemical constituent(s) in the EA fraction of LPE that inhibit HEV replication, multi-step column chromatography was employed to yield 100 mg pure compound (spicatoside A) (Fig. 4). The complete NMR spectral data for spicatoside A are presented as follows: ^1^H-NMR (pyridine-d5, 600 MHz): δ 0.82 (3 H, s, 18-CH3), 1.02 (3 H, d, J = 7.02 Hz, 27-CH3), 1.07 (3 H, d, J = 6.96 Hz, 21-CH3), 1.31 (3 H, s, 19-CH3), 1.49 (3 H, d, J = 6.36 Hz, fucose-CH3), 4.82 (1 H, d, J = 7.6 Hz, anomeric H), 5.25 (1 H, d, J = 7.6 Hz, anomeric H), 5.45 (1 H, d, J = 7.82 Hz, anomeric H), 5.53 (1 H, d, J = 5.58, H-6). ^13^C-NMR ((pyridine-d5, 600 MHz): δ 82.74 (C-1), 37.11 (C-2), 67.69 (C-3), 43.46 (C-4), 139.5 (C-5), 124.28 (C-6), 32.15 (C-7), 32.73 (C-8), 50.13 (C-9), 42.63 (C-10), 23.39 (C-11), 40.13 (C-12), 39.90 (C-13), 56.70 (C-14), 31.78 (C-15), 82.65 (C-16), 62.58 (C-17), 16.5 (C-18), 14.6 (C-19), 42.21 (C-20), 14.86 (C-21), 109.37 (C-22), 26.12 (C-23), 25.95 (C-24), 27.36 (C-25), 64.76 (C-26), 16.05 (C-27), 100.3 (fuc-1), 80.96 (fuc-2), 83.5 (fuc-3), 74.87 (fuc-4), 71.73 (fuc-5), 16.95 (fuc-6), 106.02 (xyl-1), 76.32 (xyl-2), 78.16 (xyl-3), 70.90 (xyl-4), 67.06 (xyl-5), 104.7 (glc-1), 80.96 (glc-2), 78.68 (glc-3), 72.05 (glc-4), 78.41 (glc-5), 63.08 (glc-6) (Fig. 5A).

### Spicatoside A inhibits replication of the HEV genotype 3 replicon in a concentration-dependent manner

The effects of spicatoside A, a major chemical constituent of the EA fraction of LPE (Fig. 5A), on luciferase activity of the pSHEV3-luc replicon were investigated. Huh7.5 cells transfected with transcripts from the pSHEV3-luc replicon were treated with 0, 0.5, 1 and 2 µg/ml spicatoside A, and luciferase activity of the replicon determined at four days after transfection. At doses of 0.5, 1 and 2 µg/ml, spicatoside A significantly reduced the luciferase activity of the pSHEV3-luc replicon (Fig. 5B). Our analyses yielded an estimated 50% inhibitory concentration (IC_50_) value of spicatoside A against pSHEV3-luc replicon of 0.469 ± 0.06 µg/ml (Fig. 5B). In addition, the effects of spicatoside A on replication of HEV genotype 3 strain 47832 were investigated using qRT-PCR (data not shown). Similar to data obtained with the pSHEV3-luc replicon, spicatoside A interfered with replication of HEV genotype 3 strain 47832c with an estimated IC_50_ of 0.727 ± 0.15 µg/ml.

### Spicatoside A inhibits replication of HEV genotype 3 strain 47832c and expression of HEV ORF2 *in vitro*

**Figure 6:**
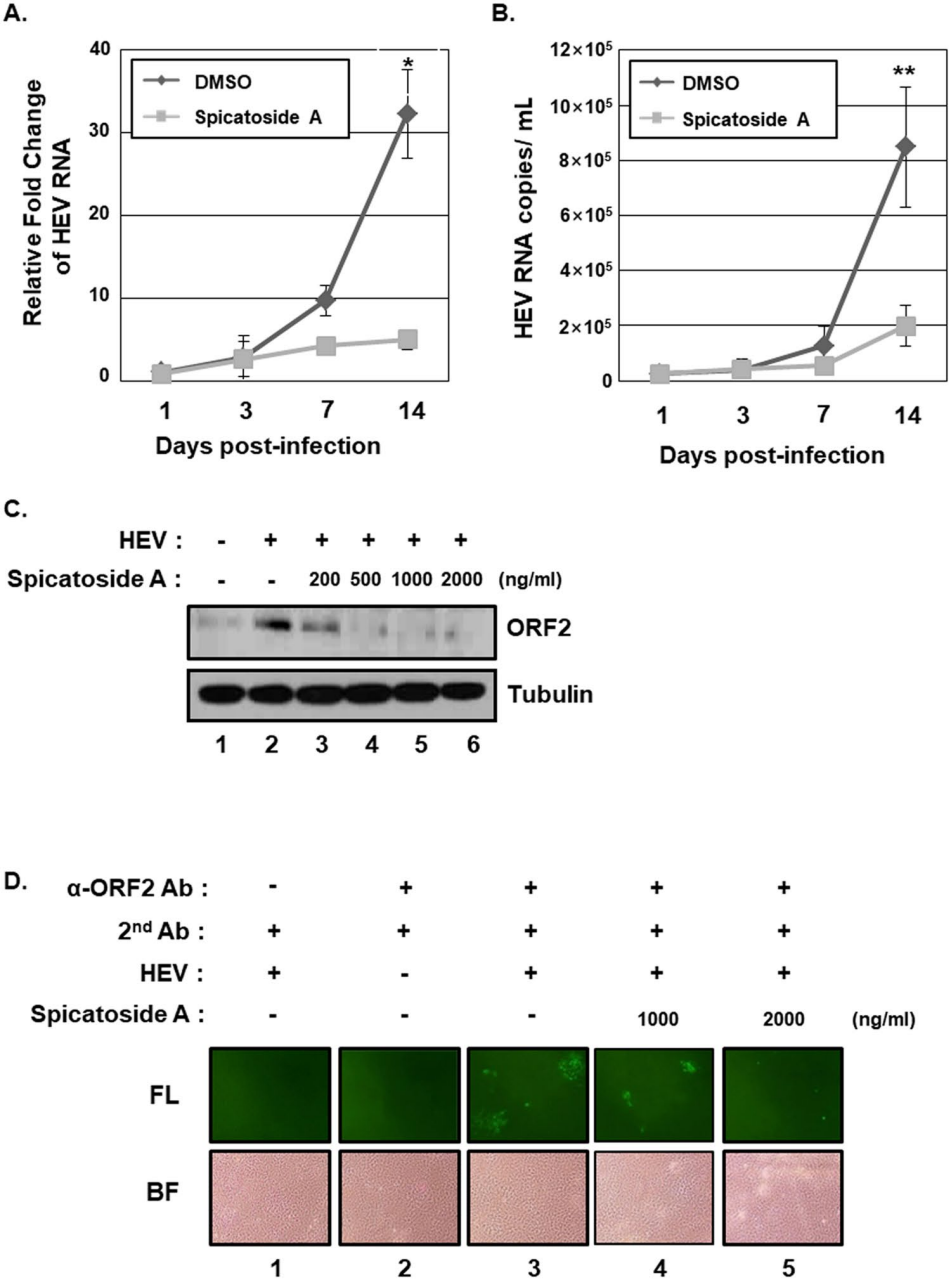
Spicatoside A inhibits replication of the HEV genotype 3 strain 47832c. A549 cells were inoculated with cell culture supernatants containing HEV genotype 3 strain 47832c and incubated at room temperature for 1 h, followed by treatment with either DMSO or spicatoside A. Cells were re-treated with either DMSO or spicatoside A every 3 d after the initial treatment. At 1, 3, 7 and 14 d after inoculation, (**A**) the relative amounts of HEV RNA in cells and (**B**) the amounts of HEV RNA copies in culture media were determined via qRT-PCR using primers specific for HEV ORF1. Relative amounts of cell-associated HEV RNA in the presence of DMSO 1 d after inoculation was defined as 1. The data shown here represent three independent experiments. At 14 d after inoculation, expression of HEV ORF2 capsid protein was determined via (**C**) western blot and (**D**) immunofluorescence microscopy. BF, bright-field microscopic image; FL, fluorescence microscopic image. *P*-values were determined by unpaired two-tailed Student’s *t*-tests (significant at *P < 0.05; **P < 0.01).

The inhibitory effects of spicatoside A against HEV replication were further established by inoculating A549 cells with HEV genotype 3 strain 47832c and treating with 2 µg/ml spicatoside A. At 1, 3, 7 and 14 d after inoculation, HEV copy numbers in cells and culture media were estimated using qRT-PCR (Fig. 6A,B). Our results showed a 32.3-fold increase in the amount of HEV RNA in DMSO-treated control cells at 14 d after inoculation (Fig. 6A). Interestingly, in cells treated with spicatoside A, the increase in HEV RNA was 15.4% lower than that in the DMSO treatment group (Fig. 6A). In addition, the increased in HEV RNA in the culture media of cells treated with spicatoside A was 23.3% lower than that in the DMSO treatment group (Fig. 6B).

To further confirm the effects of spicatoside A on replication of HEV genotype 3 strain 47832c, western blot and immunofluorescence microscopy were applied to analyze expression of the HEV ORF2 capsid protein. A549 cells inoculated with HEV genotype 3 strain 47832c were treated with different concentrations of 0, 0.2, 0.5, 1 and 2 µg/ml spicatoside A. At 14 d after inoculation, ORF2 was robustly expressed in DMSO-treated cells inoculated with HEV genotype 3 strain 47832c (Fig. 6C, compare lane 2 with 1). Notably, treatment with spicatoside A significantly reduced ORF2 expression in inoculated cells compared with vehicle controls in a dose-dependent manner (Fig. 6C, compare lane 2 with lanes 3 to 6). Consistent with western blot, down-regulation of HEV ORF2 protein was observed by immunofluorescence microscopy (Fig. 6D, compare lane 3 with lanes 4 and 5). The collective data clearly demonstrate that spicatoside A effectively inhibits HEV replication and ORF2 expression.

### Effects of Spicatoside A on viability of Huh7.5 and A549 cells

**Figure 7:**
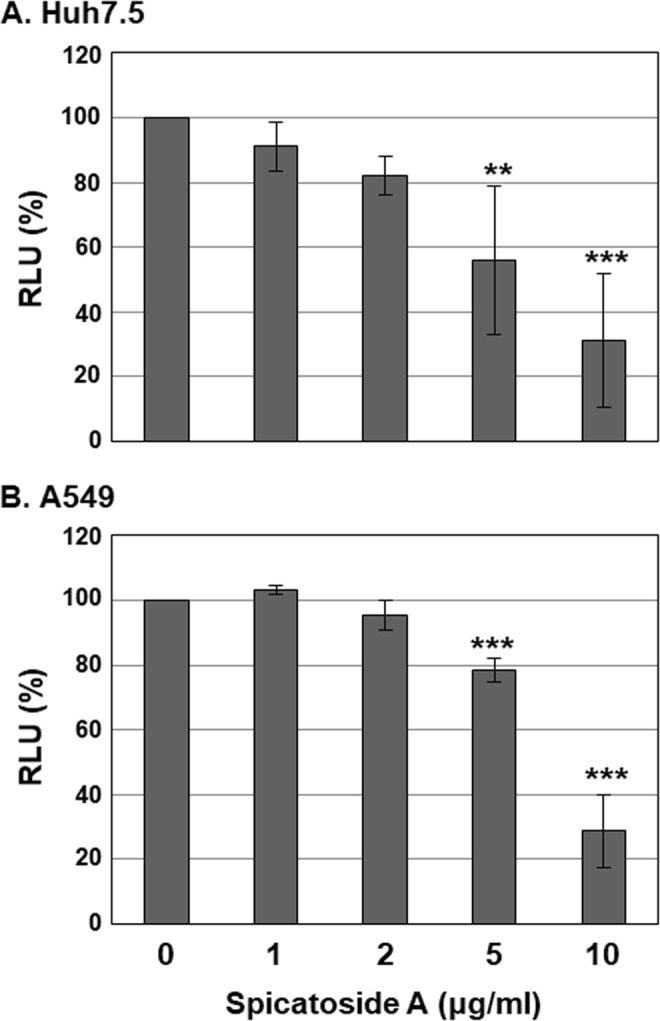
Effects of spicatoside A on cell viability. (**A**) Huh7.5 or (**B**) A549 cells were treated with varying concentrations of spicatoside A (0, 1, 2, 5 and 10 µg/ml). Cells were re-treated with spicatoside A every 3 d after the initial treatment. Huh7.5 and A549 cell viability was determined at 4 and 14 d, respectively, after treatment using the CellTiter-Glo Luminescent Cell Viability Assay. Relative luciferase activity was calculated by reference to RLU of the 0 µg/ml LPE treatment group and defined as 100%. *P*-values were determined by unpaired two-tailed Student’s *t*-tests (significant at **P < 0.01; ***P < 0.001). The data shown here represent five independent experiments.

To determine whether spicatoside A exerts cytotoxic effects, we treated Huh7.5 and A549 cells with the compound at concentrations of 0, 1, 2, 5 and 10 µg/ml and assessed cell viability. After a 4 d treatment period, spicatoside A at 5 and 10 µg/ml doses exerted significant cytotoxic effects against Huh7.5 cells inducing reduced viability to 55.9% and 31%, respectively (Fig. 7A). In A549 cells, spicatoside A had no cytotoxic effects up to 5 µg/ml after 14 d treatment (Fig. 7B) while, at 10 µg/ml spicatoside A, viability was significantly reduced to 28.6% (Fig. 7B). The IC_50_ values of spicatoside A for inhibition of Huh 7.5 and A549 cell viability were determined as 7.169 ± 9.64 and 7.486 ± 0.28 µg/mL, respectively. HEV genotype 3 strain 47832c infection exerted no additional or synergistic cytotoxic effects against A549 cells with spicatoside A (data not shown). In view of the finding that spicatoside A inhibits HEV replication and ORF2 expression at concentrations that exert no cytotoxic effects (<2 µg/ml), we propose that the antiviral activity of spicatoside A is not attributable to cytotoxicity.

## Discussion

*L. platyphylla*, a perennial plant distributed throughout Korea, Japan, and China, is reported to exert various biological activities against chronic human diseases^26,29,31,32^. In the present study, we determined the effects of LPE and its solvent fractions on HEV infection based on experiments with the HEV genotype 3 pSHEV3-luc replicon. In each case, EA, *n*-BuOH and ddH_2_O fractions of LPE were more effective inhibitors of the pSHEV3-luc replicon than LPE itself. These individual solvent fractions may contain higher concentrations of bioactive compounds against HEV than the total extract. Additionally, it is possible that bioactive compounds with anti-HEV activity in LPE counteract each other. The EA fraction showed greater efficacy against HEV infection relative to other solvent fractions and inhibited replication of both the pSHEV3-luc replicon and HEV strain 47832c without adversely affecting host cell viability. Further analysis of the EA fraction via anti-HEV activity-guided fractionation and multi-column chromatography led to the isolation of spicatoside A.

Spicatoside A is a steroidal saponin isolated from the tubers of liriopogons, cultivated genera of Liriope and Ophiopogon^33^. Various biological activities of spicatoside A have been identified to date^34^. For example, spicatoside A interferes with lipopolysaccharide (LPS)-induced activation of extracellular signal-regulated kinases (ERK1/2), p38 mitogen-activated protein kinase (MAPK), c-Jun N-terminal kinase (JNK) and NF-κB^35^. In addition to anti-inflammatory properties, spicatoside A exhibits anti-asthma, anti-osteoclastogenesis and anti-cancer activities^34^. The possibility that other bioactive compounds in LPE additionally interfere with HEV replication cannot be discounted. Considering that the *n*-BuOH and ddH_2_O fractions of LPE also inhibited replication of the pSHEV3-luc replicon, the potential bioactive compounds in these fractions warrant further investigation.

*L. platyphylla* roots are suggested to contain a bioactive compound that inhibits HBV viral promoter activity by interfering with NF-κB, but not AP-1 activity^27^. Since spicatoside A inhibits nuclear translocation of NF-κB in LPS-treated RAW264.7 macrophages^35^, one possibility is that NF-κB inhibitory activity affects replication of HEV. However, HEV utilizes ORF2 and ORF3 to inhibit NF-κB activity and thereby evade the host innate immune response^9,36^. Thus, it is likely that spicatoside A employs mechanism(s) other than NF-κB inhibition to suppress HEV replication, which will be the focus of future studies.

## Materials and Methods

### Cells, viruses and plant materials

The human hepatocellular carcinoma cell line Huh 7.5 was kindly provided by Dr. Charles M. Rice (Rockefeller University, New York, NY, USA) and cultured in Dulbecco’s Modified Eagle Medium (DMEM; Hyclone, Logan, UT) supplemented with 10% fetal bovine serum (FBS) and 1% penicillin/streptomycin (P/S). The human adenocarcinoma cell line A549 was purchased from the Korean Cell Line Bank. A549 cells were cultured in Minimum Essential Medium (MEM; Hyclone) supplemented with 10% FBS and 1% P/S. HEV strain 47832c was a kind gift from Dr. Reimar Johne (Federal Institute for Risk Assessment, Berlin, Germany)^30^. For inoculation with HEV 47832c, A549 cells were washed three times with PBS, treated with a culture supernatant containing HEV (5 genome copies/cell) and incubated at room temperature for 1 h. The supernatant was removed and replaced with medium supplemented with 5% FBS and 0.5% P/S, followed by cell incubation for 14 d. Dried root of *Liriope platyphylla* was purchased from herbal markets in Seoul, South Korea. The sample was identified by Dr. Sun Yeou Kim (College of Pharmacy (COP), Gachon University, Incheon, Korea). The plant material was preserved and a voucher specimen (KSY-SYJ1001) deposited in the herbarium of COP.

### Extraction, partition and isolation of spicatoside A from *Liriope platyphylla* root

The dried material (20 kg) was crushed to powder, extracted with 60 L of 70% ethanol at room temperature for 72 h and concentrated using a rotary evaporator resulting in 290 g ethanol extract. The ethanol extract was suspended in ddH_2_O and partitioned using the liquid partitioning technique, resulting in the following fractions: ethyl acetate (EA, 20 g), butanol (BuOH, 30 g) and ddH_2_O (238 g). Dried fractions were maintained at 4 °C before experimental use. Based on anti-HEV activity, the EA fraction was selected for further isolation using a silica gel column with a mobile phase chloroform and methanol gradient (9:1 → 0:10). Thirteen sub-fractions were obtained, and based on anti-HEV activity, sub-fraction 10 was selected for further fractionation leading to isolation of a compound (100 mg). Thin-layer chromatography (TLC) was conducted using EA: MeOH: water (100:13.5:10). To identify the isolated compound, nuclear magnetic resonance (NMR) spectroscopy was performed.

### HEV replicon, *in vitro* transcription, transfection and luciferase-reporter assay

A genotype 3 swine HEV wild-type (pSHEV3) and *Renilla* luciferase (pSHEV3-luc) replicons were kindly provided by Dr. Xiang-Jin Meng (Virginia Polytechnic Institute and State University, Blacksburg, VA, USA)^37^. The transfection control firefly luciferase-pcDNA3 (luc-pcDNA3) plasmid was a gift from William Kaelin (Addgene plasmid # 18964)^38^. pSHEV3-luc plasmids were linearized by treatment with *Xba*I (New England BioLabs, Ipswich, MA) and firefly luciferase plasmid linearized with *Bgl*II (New England BioLabs). Capped RNA transcripts from pSHEV3-luc and luc-pcDNA3 plasmids were generated using the mMESSAGE mMACHINE T7 kit (Thermo Fisher Scientific, Waltham, MA) according to the manufacturer’s instructions. Huh 7.5 cells were transfected with capped RNA transcripts produced from pSHEV3-luc (0.34 μg/10^4^ cells) and luc-pcDNA3 (0.17 μg/10^4^ cells) plasmids using DMRIE-C reagent (Invitrogen, Carlsbad, CA) in keeping with the manufacturer’s instructions. Luciferase assays were performed using the Dual-Luciferase Reporter Assay System (Promega, Madison, WI) according to the manufacturer’s protocol.

### Quantitative RT-PCR (qRT-PCR)

Quantitative analysis of cell-free and cell-associated HEV RNA was performed using qRT-PCR as previously described^24,30,39^. HEV RNA was isolated using TRI reagent (Molecular Research Center, Inc., Cincinnati, OH) and reverse-transcribed to cDNA using a TOPscript™ cDNA Synthesis kit (Enzynomics, Daejeon, Korea) as described by the manufacturer. Real-time quantitative polymerase chain reactions (PCRs) were performed using 1X HOT FIREPol EvaGreenq PCR Mix Plus (Solis BioDyne, Tartu, Estonia) according to previous reports^24,30^. The primer sequences used for amplification were as follows: HEV ORF1, 5′-CCTGTTAGTGACATTTGGGTGT-3′ (forward) and 5′-AGACCTTTGCCCCATCCGGATA-3′ (reverse); GAPDH, 5′-CATGAGAAGTATGACAACAGCCT-3′ (forward) and 5′-AGTCCTTCCACGATACCAAAGT-3′ (reverse). The amounts of HEV RNA in culture media were determined according to the method previously described^39,40^. An external standard curve was generated by using *in vitro* transcribed RNA from pSHEV3.

### Western blot analysis

Cells were harvested, fractionated and transferred onto nitrocellulose membrane, as described previously^41^. Antibodies to HEV ORF2 and tubulin were purchased from Millipore (Billerica, MA) and Sigma-Aldrich (St. Louis, MO), respectively. Secondary peroxidase-labeled anti-mouse immunoglobulin G antibodies and enhanced chemiluminescence detection reagent were purchased from (Amersham Biosciences (Piscataway, NJ) and Pierce (Rockford, IL), respectively.

### Immunofluorescence assay

Fourteen days after inoculation, A549 cells were washed three times with phosphate-buffered saline (PBS) for 5 min. After washing, cells were fixed with 80% acetone at −20 °C for 10 min and blocked with 1X PBS containing 0.5% BSA for 30 min. Subsequently, cells were rinsed with PBS and stained with mouse-HEV ORF2 antibody (MAB8002) (1:200 dilution; Millipore, Billerica, MA). After overnight incubation, cells were washed with PBS and stained with fluorochrome-conjugated anti-mouse IgG secondary antibody (4408) (1:200 dilution; Cell Signaling, Beverly, MA) at 37 °C for 1 h. Finally, cells were washed and counterstained with 4,6-diamidino-2-phenylindole (DAPI; Vector Laboratories, Burlingame, CA). Fluorescence was examined and images analyzed using an inverted Nikon TS100-F fluorescence microscope (Tokyo, Japan) equipped with a digital camera and Nikon NIS-Elements microscope imaging software.

### Cell viability assay

Cell viability was analyzed using the Cell Titer-Glo Luminescent Cell Viability Assay (Promega, Madison, WI) as described by the manufacturer.

### Statistical analysis

Data are presented as means ± standard deviations (SD). The significance of differences between two means was determined with the Student’s t-test. *P*-values were determined by unpaired two-tailed Student’s *t*-tests. *P*-values < 0.05 were considered statistically significant. Half-maximal inhibitory concentration (IC_50_) values were calculated using GraphPad Prism 7 (Graphpad Software, San Diego, CA).

## Supplementary information

Supplementary Figure S1

## Data Availability

All data and materials supporting the conclusions are described and included in this manuscript.
