# Supplementary material for: Spicatoside A derived from Liriope platyphylla root ethanol extract inhibits hepatitis E virus genotype 3 replication in vitro
Source: Sci Rep. 2019 Mar 13;9:4397. doi: 10.1038/s41598-019-39488-5 (PMC6416393; doi:10.1038/s41598-019-39488-5)
Supplement: Supplementary file 1 — Supplementary Figure S1 [file 41598_2019_39488_MOESM1_ESM.pdf]

Supplementary Figure

Article in *Scientific Reports*

Spicatoside A derived from *Liriope platyphylla* root ethanol extract inhibits hepatitis E virus genotype 3 replication *in vitro*

Gayoung Park, Amna Parveen, Jung-Eun Kim, Kyo Hee Cho, Sun Yeou Kim, Bang Ju Park, Yoon-Jae Song

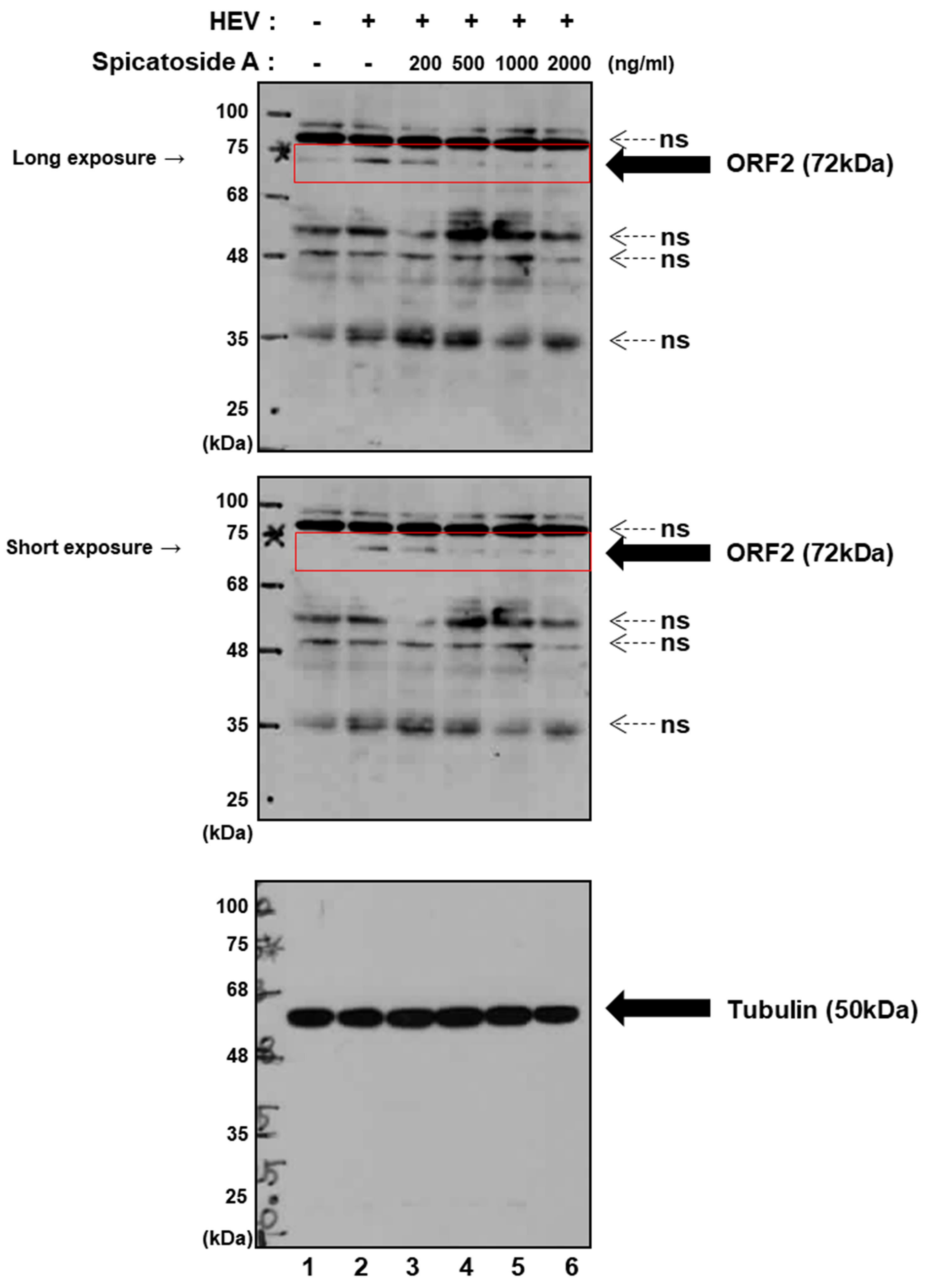

Supplementary Figure S1. Spicatoside A inhibits expression of HEV ORF2 capsid protein. A549 cells were inoculated with cell culture supernatants containing HEV genotype 3 strain 47832c and incubated at room temperature for 1 h, followed by treatment with either DMSO or spicatoside A. Cells were re-treated with either DMSO or spicatoside A every 3 d after the initial treatment. At 14 d after inoculation, expression of HEV ORF2 capsid protein was determined via western blot (ns, non-specific).
